# COVID-19 (SARS-CoV-2) lymphocyte responses are associated with inflammatory biomarkers in total joint replacement surgery candidates pre-operatively

**DOI:** 10.1186/s13018-021-02563-7

**Published:** 2021-06-30

**Authors:** Marco S. Caicedo, Vianey Flores, Alicia Padilla, Samelko Lauryn, Joshua J. Jacobs, Nadim J. Hallab

**Affiliations:** 1 Orthopedic Analysis, LLC, Chicago, IL 60612 USA; 2 Department of Orthopedic Surgery, Rush University Medical Center, Chicago, IL 60612 USA; 3 Department of Immunology, Rush University Medical Center, Chicago, IL 60612 USA

**Keywords:** Total joint arthroplasty, COVID-19, Antibodies, Lymphocytes, LTT

## Abstract

**Background:**

Recent studies indicate that, in addition to antibody production, lymphocyte responses to SARS-CoV-2 may play an important role in protective immunity to COVID-19 and a percentage of the general population may exhibit lymphocyte memory due to unknown/asymptomatic exposure to SARS-CoV-2 or cross-reactivity to other more common coronaviruses pre-vaccination. Total joint replacement (TJR) candidates returning to elective surgeries (median age 68 years) may exhibit similar lymphocyte and/or antibody protection to COVID-19 prior to vaccination

**Methods:**

In this retrospective study, we analyzed antibody titters, lymphocyte memory, and inflammatory biomarkers specific for the Spike and Nucleocapsid proteins of the SARS-CoV-2 virus in a cohort of n=73 returning TJR candidates (knees and/or hips) pre-operatively.

**Results:**

Peripheral blood serum of TJR candidate patients exhibited a positivity rate of 18.4% and 4% for IgG antibodies specific for SARS-CoV-2 nucleocapsid and spike proteins, respectively. 13.5% of TJR candidates exhibited positive lymphocyte reactivity (SI > 2) to the SARS-CoV-2 nucleocapsid protein and 38% to the spike protein. SARS-CoV-2 reactive lymphocytes exhibited a higher production of inflammatory biomarkers (i.e., IL-1β, IL-6, TNFα, and IL-1RA) compared to non-reactive lymphocytes.

**Conclusions:**

A percentage of TJR candidates returning for elective surgeries exhibit pre-vaccination positive SARS-CoV-2 antibodies and T cell memory responses with associated pro-inflammatory biomarkers. This is an important parameter for understanding immunity, risk profiles, and may aid pre-operative planning.

**Trial registration:**

Retrospectively registered.

## Background

From the beginning of the COVID-19 pandemic, 83.5% of all elective orthopedic procedures were delayed, postponed, or canceled due to lockdowns and mitigation measures across the USA and the world [1–6]. With the number of infections and fatalities more severely affecting the age group of the general total joint replacement (TJR) population, there continues to be a lack of information on rates of asymptomatic exposure, possible immunity, and overall infection risk in patients returning to elective TJR surgeries. Current epidemiological studies demonstrate that in addition to symptomatic COVID-19 patients, a percentage of the general population exhibit COVID-19 antibody titers due to unknown/asymptomatic exposure to SARS-CoV-2 or cross-reactivity to other more common beta-coronaviruses [7, 8]. Additionally, the literature points to evidence that T lymphocytes specific to SARS-CoV-2 structural proteins play a more important role than previously anticipated in conferring immunity against COVID-19 infection [9–11]. Specifically, in cases of COVID-19 convalescent individuals, not only do they exhibit detectable titers of neutralizing antibodies, but also virus-specific T lymphocytes [9, 12–14]. There is a dearth of information on how a worldwide viral pandemic affected orthopedic patients in general, and if there may be any lasting impact on biological orthopedic implant performance, i.e., immune reactivity to implants/debris. Given the evidence of possible protective immunity to SARS-CoV-2 in a subset of the general population prior to vaccination, it remains unknown whether a subset of TJR candidates returning for primary or revision surgeries (median age 66 years) would exhibit similar lymphocyte and/or antibody protection to the SARS-CoV-2 virus. We hypothesized that prior to available vaccines, a subset of TJR candidates returning from initial months of “shelter in place” orders would exhibit either cellular immunity (lymphocyte responses) and/or detectable humoral immunity (antibody titers) to SARS-CoV-2 due to unknown asymptomatic exposure to COVID-19 or cross-reactivity to other beta-coronaviruses and that these responses would also manifest as detectable increases in inflammatory biomarkers. In a retrospective cohort of primary and revision TJR candidate patients (n=73), we analyzed specific SARS-CoV-2 nucleocapsid or spike protein lymphocyte activation/proliferation and serum IgG antibody titers specific for SARS-CoV-2 at the time of pre-operative blood work.

## Materials and methods

### Subject groups and parameters

Retrospective blinded de-identified data from a cohort of n=73 returning TJR candidates (knees and/or hips) tested for lymphocyte function with an in vitro lymphocyte transformation test (LTT) was studied (approved under Rush University IRB). A group of male (n=18) and female (n=55) returning TJR candidates referred for lymphocyte transformation testing were screened for COVID-19 antibody titers and lymphocyte reactivity to SARS-CoV-2 nucleocapsid and spike proteins during pre-op testing (Table 1); none reported having a confirmed COVID-19 positive test or being exposed COVID-19 at time of sample collection.

**Table 1:**
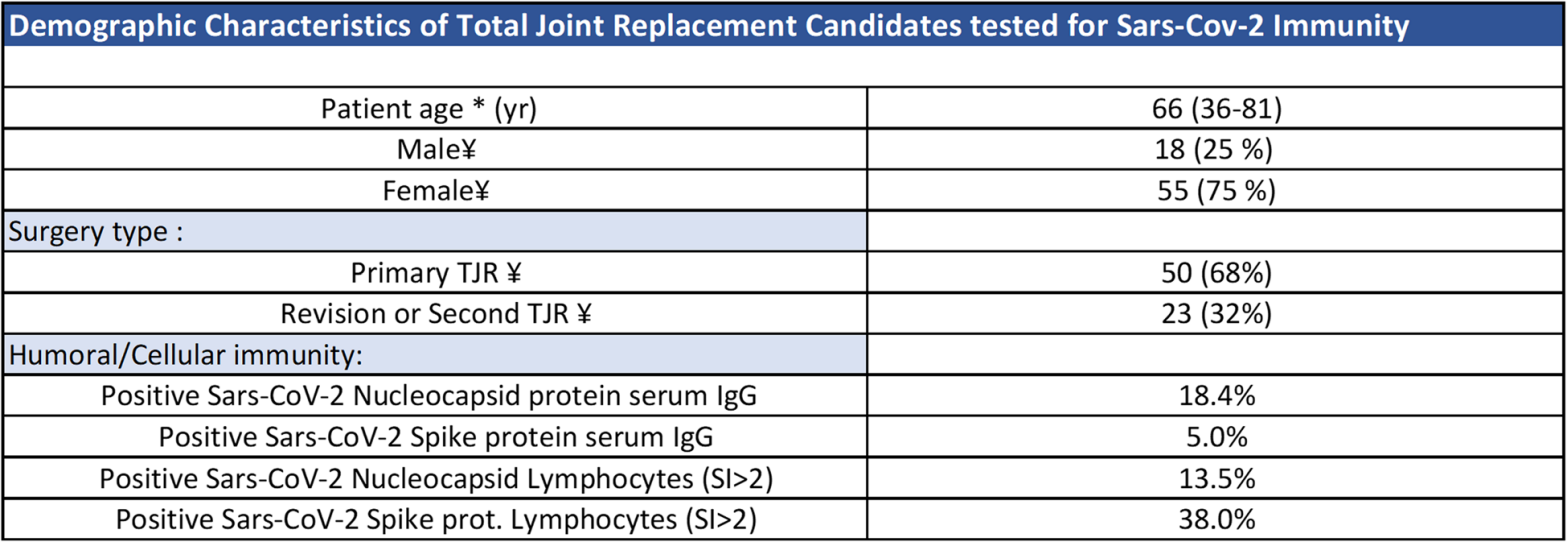
Demographics of TJR candidates tested for SARS-CoV-2 humoral and cellular immunity. *The values are given as the mean, with the range in parentheses for patient age. ¥The values are given as the number, with the percentage in parentheses

### Sample collection and lymphocyte transformation test for SARS-CoV-2

Whole blood was collected by venipuncture from TJR patients referred from healthcare facilities representative of 18 states in the nation using a specialized blood collection kit to ensure the quality of blood draw supplies and sample temperature stability during transport. All samples were transported to the testing facility priority overnight and processed within 24 h of initial collection. Peripheral blood mononuclear cells (PBMCs) were collected from 30 ml of peripheral blood by Ficoll gradient separation. Collected PBMCs (white buffy coat) were washed in PBS and re-suspended in RPMI-1640 with 10% autologous serum and cultured with 5μg/ml of SARS-CoV-2 nucleocapsid or spike protein (Miltenyi Biotec, CA) at 5% CO^2^ and 37 °C for 6 days. ^3^H thymidine was added at day 5 of culture. ^3^H thymidine incorporation in unchallenged (control) and metal-treated PBMCs was analyzed using a beta scintillation counter at day 6. A stimulation index (SI) of reactivity was calculated by dividing scintillation counts per minute of SARS-CoV-2-challenged cells over untreated controls. Results were designated as non-reactive for SI < 2, or reactive for SI > 2.

### Serum SARS-CoV-2 nucleocapsid IgG detection

#### Enzyme-linked immunoassay

Serum samples were collected from whole blood of TJR candidates. Sandwich enzyme-linked immunoassays for SARS-Cov-2 nucleocapsid (Epitope Diagnostics, CA) and spike proteins (Bethyl Labs, TX) were performed with the manufacturer’s provided buffers and protocols.

### Multiplex Luminex assays

Luminex MultiAnalyte Assay (EMD Millipore) analysis was used to detect human IL-1β, TNF-α, IL-6, IL-1RA, and IL-10 in SARS-CoV-2 peripheral blood mononuclear cell culture supernatants. Samples were collected at day 5 of culture, aliquoted, and stored at − 80 °C until time of analysis.

### Positivity rates and statistical analysis

Rate of SARS-Cov-2 nucleocapsid and spike protein IgG positivity was calculated by the percentage of TJR positive serum samples (OD > 0.3 for nucleocapsid protein and OD > 0.15 for spike protein). Rate of lymphocyte reactivity to SARS-CoV-2 nucleocapsid and spike proteins was calculated by the percent of reactive (SI > 2.0) subjects in each group. Statistical differences in pro-inflammatory cytokine production were analyzed with parametric t test reaching significance at p < 0.05.

## Results

### Rate of positive SARS-CoV-2 nucleocapsid and spike protein IgG antibodies in TJR candidates

**Fig. 1:**
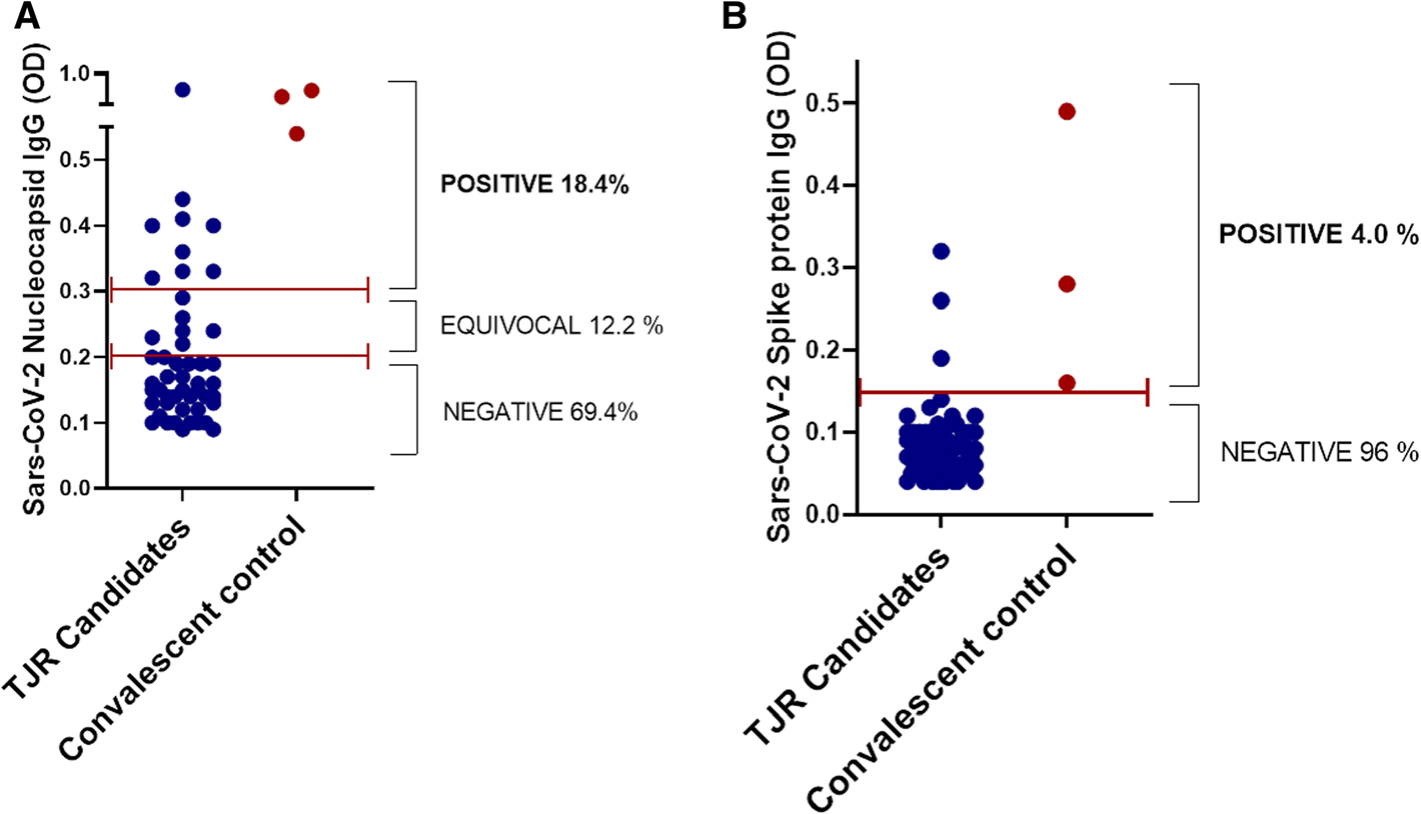
SARS-CoV-2 Antibody titers in TJR candidates. **A** Nucleocapsid protein serum IgG was measured in TJR candidates (n=49). **B** Spike protein serum IgG was measured in TJR candidates (n=64). Each dot represents each subject tested. Convalescent serum of COVID-19 confirmed cases were used as positive controls for nucleocapsid and spike protein IgG. Dotted bars indicate the threshold of antibody levels based on the manufacturer’s instructions. OD, optical density

To measure the level of serum SARS-CoV-2 nucleocapsid and spike protein antibodies (IgG) in TJR candidates, peripheral blood serum collected pre-operatively was tested via enzyme-linked immunoassay for SARS-CoV-2 nucleocapsid (n=49) and spike protein (n=65) IgG antibodies (Fig. 1). Nine (18.4%) of 49 TJR candidates exhibited elevated serum levels of SARS-CoV-2 nucleocapsid IgG while 6 (12.2%) had borderline/equivocal positive results and 69.4% of TJR candidates tested negative for SARS-CoV-2 nucleocapsid protein IgG (Fig. 1A, Table 1). Contrary to nucleocapsid IgG titers, only 3 (5%) of 65 TJR candidates exhibited positive antibody titers for spike protein IgG with the majority of patients testing negative (95%) (Fig. 1B, Table 1). Convalescent serum from polymerase chain reaction (PCR) confirmed cases of COVID-19 exhibited elevated titers of both nucleocapsid and spike SARS-CoV-2 proteins (Fig. 1).

### Rate of lymphocyte memory to SARS-CoV-2 in TJR candidates

**Fig. 2:**
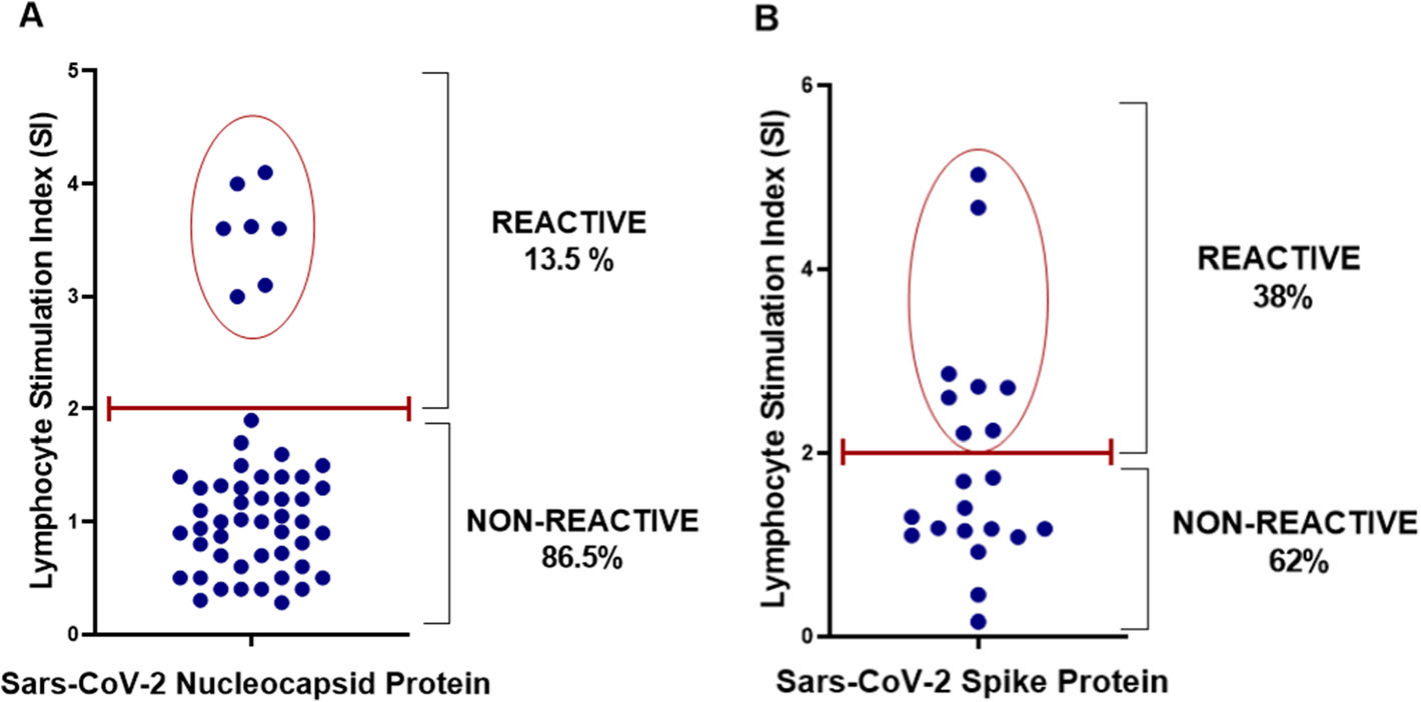
SARS-CoV-2 lymphocyte reactivity in TJR candidates. SARS-CoV-2 adaptive immunity was determined by lymphocyte transformation testing against SARS-CoV-2 nucleocapsid (**A**) and spike (**B**) proteins in TJR candidates (n=73). Lymphocyte stimulation index (SI) was achieved by normalizing nucleocapsid- and spike protein-treated lymphocyte counts per minute (cpms) to unchallenged lymphocyte controls. Each dot represents each subject tested and an SI > 2 was considered a positive reactive result

In the incidence of adaptive cellular immunity to SARS-CoV-2, TJR was compared (n=73) when tested for lymphocyte reactivity using a lymphocyte proliferation test in response to SARS-CoV-2 nucleocapsid (n=52) or spike proteins (n=21) (Fig. 2). Eight (15%) of the 52 subjects tested for lymphocyte activation/proliferation to the SARS-CoV-2 nucleocapsid protein showed reactivity with a stimulation index higher than 2-fold (SI > 2) and 44 (85%) did not exhibit any significant lymphocyte proliferation (SI < 2) to this specific protein (Fig. 2A). Additionally, eight (38%) of the 21 subjects tested for the SARS-CoV-2 spike protein exhibited lymphocyte activation/proliferation (SI > 2) while 13 (62%) showed no significant lymphocyte proliferation (SI < 2) to this specific protein (Fig. 2B). The combined overall rate of adaptive lymphocyte memory to either the SARS-CoV-2 nucleocapsid or spike proteins in TJR candidates returning to elective surgery was 21% (Fig. 2).

### Pro-inflammatory cytokine production in response to SARS-CoV-2 proteins in TJR candidates

**Fig. 3:**
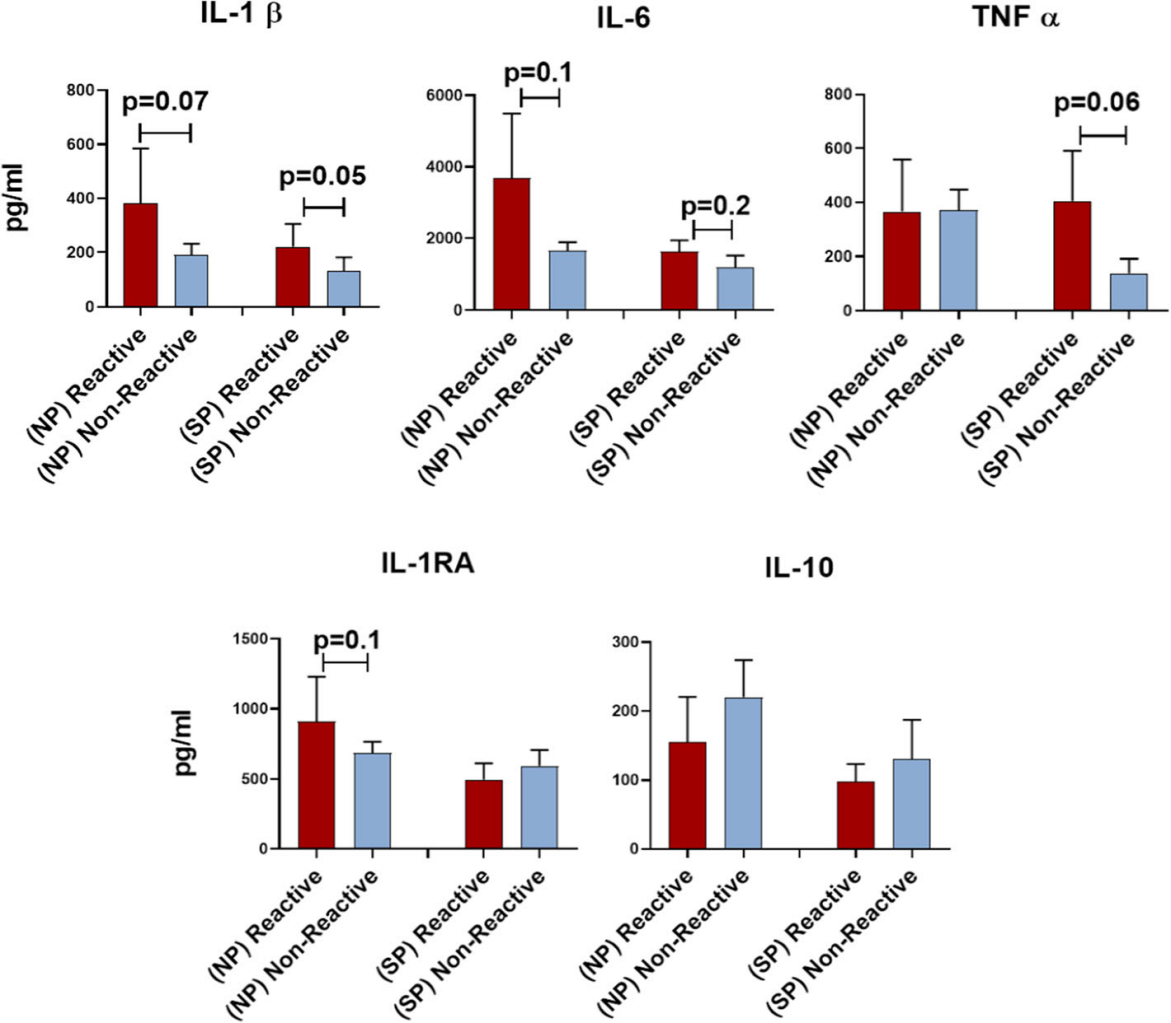
Cytokine production in response to SARS-CoV-2 nucleocapsid (NP) and spike proteins (SP) in TJR candidates. Peripheral blood mononuclear cell (PBMC) pro-inflammatory Il-1β, IL-6, TNFα and anti-inflammatory IL-10, and IL-RA were measured in response to SARS-CoV-2 nucleocapsid protein (NP) and spike protein (SP) using a multiplex analyte assay. Error bars represent the standard error of the mean for each group and p values between groups are listed

SARS-CoV-2 lymphocyte culture supernatants from TJR candidates were collected and analyzed for pro-inflammatory cytokine production at day 5 of culture. Not surprisingly, orthopedic patients with SARS-CoV-2 lymphocyte reactivity exhibited higher production of pro-inflammatory cytokines compared to those non-LTT reactive. SARS-CoV-2 nucleocapsid protein reactive lymphocytes exhibited a higher production of IL-1β (382.92 pg/ml) compared to non-reactive subjects (190.5 pg/ml) (p=0.07). Similarly, IL-6 and IL-1RA were found in greater quantities (p ≤ 0.1) in nucleocapsid reactive subjects at 3685 pg/ml and 909.82 pg/ml compared to non-reactive subjects at 1659.5 pg/ml and 684.6 pg/ml, respectively (Fig. 3A). TNFα was not higher in nucleocapsid reactive subjects (365.38 pg/ml) compared to non-reactive subjects (370.6 pg/ml). Anti-inflammatory IL-10 differences did not significantly differ, but lower concentrations in nucleocapsid reactive subjects (154.44 pg/ml) compared to non-reactive subjects were detected (219.9 pg/ml) (Fig. 3A). A similar association of pro-inflammatory cytokine production was observed for spike protein reactive subjects compared to non-reactive subjects. Spike protein reactive subjects exhibited significantly higher production of IL-1β (221.1 pg/ml, p=0.05), TNFα (405.15 pg/ml, p=0.06), and IL-6 (1632 pg/ml, p=0.2) compared to non-reactive subjects which only exhibited 132.1 pg/ml, 138.5 pg/ml, and 1194.6 pg/ml of IL-1β, TNFα, and IL-6, respectively (Fig. 3B). Non-significant differences in anti-inflammatory cytokines IL-10 (97.6 pg/ml) and ILRA (495.8 pg/ml) were observed between the reactive group compared to the non-reactive spike protein group with 130.1 pg/ml of IL-10 and 590.4 pg/ml of IL1RA (Fig. 3B).

## Discussion

Results of this study support our hypothesis that a subpopulation of returning TJR candidates have specific immunological memory to SARS-CoV-2 due to unknown asymptomatic exposure to the virus or previous exposure to other common beta coronaviruses and indicate that this immune response is associated with elevated inflammatory biomarkers in vitro. These data show that 18.5% of TJR candidates preparing for elective surgery have a significant level of IgG antibodies specific for the nucleocapsid protein of SARS-CoV-2 and another 12% exhibited borderline levels for the antibody. Surprisingly, only 5% of TJR candidates exhibited elevated levels of spike protein antibodies (IgG). This could have been the result of the more transient levels of spike protein neutralizing antibodies observed in mild or asymptomatic infections compared to the more immunogenic properties of nucleocapsid proteins [15]. While it is still unclear what specific levels of SARS-CoV-2 antibodies may confer any individual immune/protected to subsequent infection, it is remarkable that almost one in five TJR candidates likely possess some level of humoral protection. It is important to note that the higher incidence of antibodies detected was specific for the more immunogenic nucleocapsid protein of SARS-CoV-2, which is a highly conserved protein among the family of coronaviruses. However, the most significant cytokine differences between LTT reactive and non-reactive groups were to the spike protein recall response. It is possible that patients with high levels of nucleocapsid protein and low or no levels of spike protein antibodies may have been infected by SARS-CoV-2 but may experience a milder form of the disease due to immunological memory to the nucleocapsid antigen, which would be detected shortly after infection due to its abundance. Interestingly, in addition to detecting humoral (antibody) immunity to SARS-CoV-2, our data indicates that TJR candidates also exhibited specific adaptive immunity to both the nucleocapsid and spike proteins of SARS-CoV-2. Initial immune recognition of foreign antigens by lymphocytes such as CD4+ helper T cells precedes antibody production by B lymphocytes and is crucial for the development of productive humoral immunity if necessary. Using a lymphocyte transformation test modified for the detection of SARS-CoV-2 nucleocapsid and spike proteins, we found that 13.5% of TJR candidates exhibited lymphocyte reactivity to the nucleocapsid protein while 38% exhibited reactivity to the spike protein. These data are important now, given that it was obtained in the early months of the pandemic prior to vaccination and thus represents a unique data point to compare to current post-vaccine incidence, reactivity, and related implant performance. Additionally, this has important implications in understanding which patients can be considered protected/immune, given that only antibody testing is currently available to assess conferred immunity and may not reflect a complete assessment of true immune status. There are no commercially available tests that examine specific lymphocyte memory (adaptive immunity) to SARS-CoV-2, which likely is a more sensitive measure of immunity even in the absence of circulating antibodies as it is the case in other viral diseases [16]. Not surprisingly, culture supernatants from SARS-CoV-2 reactive lymphocytes exhibited higher production of potent pro-inflammatory biomarkers like IL-1β, TNFα, and IL-6 compared to non-reactive lymphocytes. This supports the notion that patients with adaptive immunological memory may mount a stronger and more productive response to SARS-CoV-2, which may render them more protected during infection [11]. The potential significant increase in inflammatory biomarkers (i.e., IL-1β and IL-6) post COVID-19 infection/resolution indicates there may be a lasting altered pre-operative immune profile/environment capable of inducing musculoskeletal symptoms and affecting orthopedic surgery post-infection and recovery [17]. This may have important ramifications to implant performance. If this association is consistent in a larger scale study, it indicates that people who have had symptomatic or asymptomatic COVID-19 may require increased surveillance/follow-up after orthopedic surgery (i.e., total joint arthroplasty). One caveat in our study is that all antibody-positive subjects, except for one, did not exhibit lymphocyte reactivity (SI < 2) and had lower pro-inflammatory cytokine production compared to antibody-negative subjects. This suggests that SARS-CoV-2 IgG serum antibodies found may be of neutralizing nature, rendering lymphocytes unable to mount a productive response in an in vitro cell culture system where the amount of antigen used is limited to the culture system and not able to overburden neutralizing antibodies. This represents an important limitation of results interpretation that may be generalizable to ex vivo measured lymphocyte responses in other assays as well.

## Conclusions

Our combined data suggests that a subgroup of TJR candidates may have a complex background of humoral and/or cellular immunological memory to SARS-CoV-2 associated with pro-inflammatory cytokine production (primarily IL-1β) which may render a certain level of protection to SARS-CoV-2. Additionally, humoral (IgG antibodies) and cellular immunity (lymphocyte memory) may not necessarily occur concomitantly but may provide protective immunity via different immune mechanisms; both of which have the potential to affect immune homeostasis and impact biologic reactivity to orthopedic implants. This is an important parameter for understanding risk profiles and may aid pre-operative planning.

## Data Availability

All data generated or analyzed during this study are included in this published article and its supplementary information files.

## Abbreviations

TJR: Total joint replacement
LTT: Lymphocyte transformation test
ELISA: Enzyme-linked immunoassay
IgG: Immunoglobulin G
COVID-19: Coronavirus disease 19
NP: Nucleocapsid protein
SP: Spike protein
